# Robust Adaptive Transmit Beamforming under the Constraint of Low Peak-to-Average Ratio

**DOI:** 10.3390/s22197278

**Published:** 2022-09-26

**Authors:** Hongtao Li, Zhoupeng Ding, Sirui Tian, Songpo Jin

**Affiliations:** 1 School of Electronic and Optical Engineering, Nanjing University of Science and Technology, Nanjing 210094, China; 2 China Electronics Technology Group Corp 54th Research Institute, Shijiazhuang 050081, China

**Keywords:** PAPR, SOCP, transmit beamforming, ASV mismatch

## Abstract

In radar detection, in order to make the beam have variable directivity, a Capon beamformer is usually used. Although this traditional beamformer enjoys both high resolution and good interference suppression, it usually leads to high sidelobe and is sensitive to array steering vector (ASV) mismatch. To overcome such problems, this study devises a novel, robust adaptive beamformer that is robust to ASV mismatch under the constraint where the sidelobe is oriented to the ground. Moreover, to make full use of the transmit power, the constraint of a low peak-to-average power ratio (PAPR) is also taken into consideration. Accordingly, this robust adaptive beamformer is developed by optimizing a transmitting beamformer constrained by ASV mismatch and low PAPR. This optimization problem is transformed into a second-order cone programming (SOCP) problem which can be efficiently and exactly solved. The proposed transmit beamformer possesses not only adaptive interference rejection ability and robustness against ASV mismatch, but also direct sidelobe control and a low PAPR. Simulation results are presented to demonstrate the superiority of the proposed approach. The proposed method can make the peak sidelobe level (PSL) level on the ground side below −30 dB.

## 1. Introduction

In modern radar systems, the adaptive array is usually used to suppress interference [1]. When conventional adaptive digital beamforming (ADBF) is used for receiving beam shaping, it can form a null in the receiving pattern [2]. However, according to the transmit–receive anisotropy of the antenna, if the transmit pattern is consistent with the receive pattern, transmission energy loss inevitably occurs due to beamforming. This leads to the dispersion of radar transmitting energy, and it is not possible to irradiate as much energy as possible to the target area of interest. As the transmitted energy is dispersed, the power of the echo is also affected. When the radar system detects low, small, and slow targets, although receiving ADBF can adaptively form a null, the strong interference brought by the ground cannot be well suppressed.

The research on ADBF technology has become comprehensive in recent years. Practically, many factors that seriously deteriorate the target detection performance cannot be ignored, including wavefront distortion, incoherent local scattering, terminal pointing errors, and antenna array calibration errors [3]. Conventional transmit beamforming has high resolution and good interference suppression, but it also leads to high sidelobe and is sensitive to array steering vector (ASV) mismatch [4,5,6]. To improve the robustness of the beamformer, several algorithms have been promoted in past decades. For example, the loading methods of Li [7], Mestre and Lagunas [8], Du [9], and Zhuang [4] are known as the diagonal loading (DL) approach, which adds a fixed identity matrix to the sample covariance matrix (SCM). However, it is difficult to determine the appropriate loading factor in different scenarios. If the DL level is chosen improperly, the SCM cannot be approximately equal to the ideal covariance matrix. Thus, the robustness of the DL beamformer is degraded. Replacing the SCM with an enhanced covariance matrix can effectively reduce the chance of SV mismatch, but the performance improvement is not obvious. With the increase in the signal-to-noise ratio (SNR), the performance of the DL algorithms is severely depressed.

The subspace approach is another technology that works against ASV mismatch. Zhang estimated noise subspace and interference subspace via constructing an interference-plus-noise covariance matrix [10]. When ASV is mismatched, this method can achieve a high resolution, but it has high computational complexity, and the performance of subspace-based beamformers degrades drastically at low SNRs, where the signal subspace may be corroded by the noise subspace. Moreover, it requires that the dimension of the signal-plus-interference subspace be exactly known and be much lower than the number of sensors, which means it needs large snapshots and has strict requirements regarding the amount of interference. In order to maximize the signal-to-interference-to-noise ratio (SINR), Vouras proposed a broadband array robust transmit nulling (RTN) algorithm [11]. This method deduces the functional relationship between SINR and the frequency integral. The function is solved according to the conjugate iterative algorithm so as to obtain the optimal weight vector. Since the method requires several iterations to obtain an optimal solution, the real-time performance is poor [12]. Considering that an insufficient number of snapshots and high computation complexity result in the performance degradation of beamformers, a fast and robust adaptive beamforming algorithm was proposed by Jun [13]. This method formulates the weight vector as a linear combination of the training samples and the signal steering vector. Regularization techniques are also utilized to suppress the excessive variation of the combination vector. This study reduces the computation complexity dramatically.

A new robust adaptive beamforming (RAB) method was proposed recently. It eliminates the influence of the desired signal by reconstructing the interference-plus-noise covariance (INC) matrix. In the literature [14], Gu proposed a method based on the INC matrix reconstruction and steering vector estimation. It integrates the angular sectors separated in the signal of interest (SOI) direction, reconstructs the INC matrix based on the Capon spectrum, and, finally, estimates the steering vector of the desired signal by solving a quadratically constrained quadratic programming (QCQP) problem. Compared with conventional RAB technology, this method has excellent performance, but this kind of method cannot effectively solve the array calibration error. Furthermore, the computational complexity is comparatively large because of the integral operation. To achieve a larger array aperture, the coprime array was studied in [15]. In [15], the coprime array was decomposed into a pair of sparse uniform linear subarrays and processed signals separately. According to the property of coprime integers, the direction of arrival (DOA) can be uniquely estimated. The estimated DOAs and their corresponding power were utilized to reconstruct the INC matrix and estimate the signal steering vector.

Joint radar and communications on a single platform can reduce the cost of the platform, share spectrums, and enhance performance via the cooperation of radar and communications [16,17,18]. Joint transmit beamforming has been recently studied. As a special beamformer, a radar beamformer can learn from the technology of general beamformers to a certain extent. A beam codeword is a set of analogue, phase-shifted values applied to the antenna elements, forming an analogue beam. On the one hand, beamforming based on depth learning is proposed to utilize the channel state information. In [19], a mitigation method for adversarial attacks against proposed 6G machine learning models was proposed for millimeter wave (mmWave) beam prediction using adversarial learning. On the other hand, beamforming without machine learning has also been widely studied. By encoding a communication message into a radar waveform, radar can function as an information embedding system [20]. In [21,22], array transmit beamforming was designed to synthesize radar and communication. In this vein, an alternating-projection, two-stage iterative method was developed and used to design a set of physical, multi-function waveforms with a common antenna array. This method takes into account waveform synthesis at the main lobe but cannot suppress the azimuth sidelobe of the radar transmit beampattern. Liu [16] proposed a method to minimize the interference power at communication receivers. However, this algorithm does not consider the SINR of each user. In [23], a method for information embedding using a time-modulated array was proposed. The phases of the transmit weight vectors are adjusted from pulse to pulse in order to introduce variations in the sidelobe levels (SLLs) towards the intended communication receiver. However, it is difficult to design multiple transmit power distribution patterns with the same main lobe for time-modulated arrays. Hassanien developed a new technique with two weight vectors for dual-function radar communication (DFRC) [24]. Sidelobe control of the transmit beamforming in tandem with waveform diversity enables communication links using the same pulse radar spectrum. This method assumes that the desired ASV mismatch is not mismatched. When array signals are only used for radar detection (such as monostatic array radar), transmit beamforming can design waveforms according to the radar environment so as to avoid interference as much as possible.

In this paper, a new transmitting beamformer is proposed. Considering the disadvantages of conventional beamformers, such as high sidelobe and sensitivity to ASV mismatch, the direction of the sidelobe facing the ground is limited, and it is robust under ASV mismatch. In addition, to make full use of the amplified power of the transmitter, better suppress the large-area interference, and irradiate the radar energy to the target area as much as possible, the constraint of a low peak-to-average power ratio (PAPR) is also considered. Simulation results show the superiority of this method. Generally, the major contributions of this paper include:

(1) The maintenance of the main lobe performance for radar beamforming;
(2) The suppression of the sidelobe of radar beampattern;
(3) Introduction of the PAPR constraint to improve radar detection performance.

The rest of this paper is organized as follows. In Section 2, the signal model of the array radar is established. The beamformer under ASV mismatch, the low sidelobe constraint, and the low PAPR constraint is derived. The final form of the beamformer is proposed at the end of this section. Simulation results and performance analyses are provided in Section 3. Conclusions are summarized in Section 4.

## 2. The Proposed Method

### 2.1. The Array Radar Transmit Signal Model

**Figure 1:**
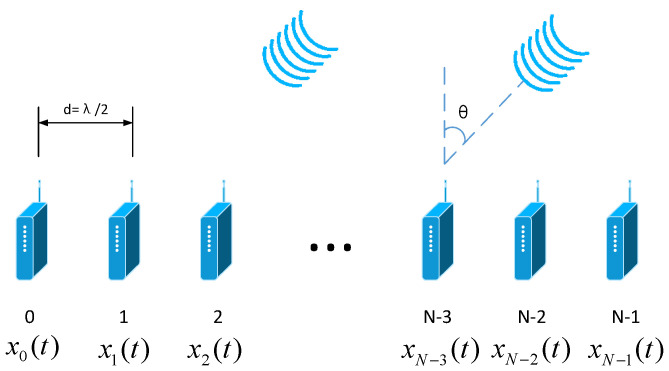
Uniform liner array radar transmit signal model.

A uniform linear array (ULA) composed of N isotropic antennas with inter-element spacing d is considered, as shown in Figure 1.

A plane wave with a λ wavelength impinges on the array from angle θ. If the transmitted signals are narrowband, the sensor-sampled signal $x_{n}\left(i\right)$, n=0,1,⋯,N−1 is emitted by the *n*th array sensor. The transmit beampattern can be expressed as

(1) $$\begin{array}{cc} G\left(\theta \right) & =\sum_{n=0}^{N-1}\omega_{n}x_{n}\left(i\right)\exp\left(-j\frac{2\pi }{\lambda }nd\sin\theta \right)\\ & =a^{H}\left(\theta \right)\left[\omega ⊙x\left(i\right)\right] \end{array}$$

with

(2) $$x\left(i\right)={\left[x_{0}\left(i\right),x_{1}\left(i\right),\cdots ,x_{N-1}\left(i\right)\right]}^{T}$$

(3) $$\omega ={\left[\omega_{0},\omega_{1},\cdots ,\omega_{N-1}\right]}^{T}$$

(4) $$a\left(\theta \right)={\left[1,\exp\left(-j\frac{2\pi }{\lambda }d\sin\theta \right),\cdots ,\exp\left(-j\frac{2\pi }{\lambda }\left(N-1\right)d\sin\theta \right)\right]}^{T}$$

where ${\left(\cdot \right)}^{H}$ and ${\left(\cdot \right)}^{T}$ represent the conjugate transpose and transpose operators, respectively; $\omega_{n}$ is the complex transmit weight for the nth array element with ω, the transmit weight vector (TWV); $a\left(\theta \right)$ is the ASV at direction θ; and ⊙ symbolizes the Hadamard product. Without a loss of generality, the probing signal $x_{n}\left(i\right)$ is assumed to be the same for all antennas for the array-phased radar. As a result, the transmit beampattern in (1) can be rewritten as

(5) $$G\left(\theta \right)=a^{H}\left(\theta \right)\omega$$

According to the principle of the transceiver reciprocity, ω can be designed by utilizing the received signals.

It is well known that conventional adaptive beamformers, e.g., the minimum variance distortionless response (MVDR) beamformer [25], minimize array output power subject to a unity gain at the desired direction $\theta_{0}$. That is,

(6) $$\begin{array}{l} \underset{\omega }{\min}\;\omega^{H}R\omega \\ s.t.\;a^{H}\left(\theta_{0}\right)\omega =1 \end{array}$$

where $a^{H}\left(\theta_{0}\right)$ is the ASV of the desired target, and R is the covariance matrix of the array snapshot for the received signals.

(7) $$R=E\left\{y\left(i\right)y^{H}\left(i\right)\right\}$$

where $E\left\{\cdot \right\}$ is the mathematical expectation. $y\left(i\right)$ is the observation vector composed of the components of signal, interference, and noise and is defined as

(8) $$y\left(i\right)={\left[y_{0}\left(i\right),y_{1}\left(i\right),\cdots ,y_{N-1}\left(i\right)\right]}^{T}$$

The optimal weight vector is the solution of (6).

(9) $$\omega_{\mathrm{opt}}=\alpha R^{-1}a\left(\theta_{0}\right)$$

where α is the normalization factor that does not affect the output SINR. In practical applications, R is not exactly available and is usually obtained from a finite set of samples, as

(10) $$\hat{R}=\frac{1}{L}\sum_{i=1}^{L}y\left(i\right)y^{H}\left(i\right)$$

where L is the number of training snapshots. In this case, the beamformer weight vector can be given by

(11) $$\omega_{\mathrm{SMI}}={\hat{R}}^{-1}a\left(\theta_{0}\right)$$

which is commonly referred to as the sample matrix inversion (SMI) beamformer. It is well known that the SMI beamformer can provide rapid convergence of the output SINR to the optimal value. However, this kind of method does not consider the energy distribution during the launch, that is, it irradiates the launch energy to the target area as much as possible without weakening the radar detection power rather than just forming a zero null for the interference. Therefore, the PAPR of the TWV should not be too high to make better use of transmission energy. On the other hand, the SMI algorithm cannot provide sufficient robustness against ASV mismatch, which is due to the errors in array antenna spacing, channel amplitude, and channel phase. Moreover, $\omega_{\mathrm{SMI}}$ cannot provide flexible sidelobe suppression according to actual requirements.

### 2.2. The Proposed Method

Practically, a radar system that aims to detect low altitude, slow-speed, small targets should transmit all energy into the target area in an ideal condition so the receive module can obtain a stronger SNR and a better performance. However, if the radar transmitter is amplitude weighted, the radar emission energy is weakened. Considering that a radar transmitter amplifier usually works in a saturated state and cannot implement the amplitude modulation on the waveform, the emitted signal is often required to be unimodular or have a low PAPR. The low PAPR constraint is the relaxation of the unimodular constraint so that the radar transmitter can transmit as much energy as possible to the target area. With the above under consideration, we impose a low PAPR constraint on the TWV. According to the definition, the constraint on the PAPR of the TWV can be expressed as

(12) $$r_{\mathrm{PAPR}}=\frac{\underset{i=1,2,\ldots ,N}{\max}{\left|\omega_{i}\right|}^{2}}{{\left\|\omega \right\|}^{2}/N}=N\frac{\underset{i=1,2,\ldots ,N}{\max}{\left|\omega_{i}\right|}^{2}}{{\left\|\omega \right\|}^{2}}\leq \zeta$$

where ζ is the system PAPR tolerance.

Furthermore, there may be mismatches in the array element position, channel amplitudes, and channel phases, which leads to ASV mismatch. The SMI beamformer in (11) is highly sensitive to ASV mismatch. In a mismatch case, the actual beampattern is distorted so that the spatial filtering capability is reduced, and other substantial performance degradations are caused.

Let the position of the *n*th element of the array be $p_{n}$, and let the real position of the array element be

(13) $${\hat{p}}_{n}=p_{n}+\Delta p_{n}$$

The ideal weighting vector of the beamformer is

(14) $$\omega_{n}=g_{n}e^{j\varphi_{n}}$$

where $g_{n}$ is the ideal amplitude weighting coefficient, and $\varphi_{n}$ is the ideal phase weighting coefficient. In practice, if the error is included in the weighted value, the actual amplitude and phase weighting coefficients are

(15) $${\hat{g}}_{n}=g_{n}+\Delta g_{n}\;,\;{\hat{\varphi }}_{n}=\varphi_{n}+\Delta \varphi_{n}\;$$

Assume that $\Delta g_{n},\Delta \varphi_{n},\Delta p_{n}(n=1,2,\cdots ,N)$ is a statistically independent, zero-mean, Gaussian, random variable.

The actual beam magnitude response is

(16) $$\hat{F}(\theta )={\hat{\omega }}^{H}\hat{a}(\theta )=\sum_{i=1}^{N}{\hat{g}}_{n}e^{j({\hat{\varphi }}_{n}-2\pi {\hat{p}}_{n}sin(\theta )/\lambda )}$$

where $\hat{\omega }$ and $\hat{a}(\theta )$ represent the actual weight vector and the actual ASV.

According to (16), with tiny mismatches in the above-mentioned cases, the expectation of the actual beam magnitude response is

(17) $$\begin{array}{c} E\left\{{\left|\hat{G}(\theta )\right|}^{2}\right\}={\left|G(\theta )\right|}^{2}\exp\left[-(\sigma_{\varphi }^{2}+\sigma_{\lambda }^{2})\right]+(\sigma_{\varphi }^{2}+\sigma_{\lambda }^{2}+\sigma_{g}^{2})\sum_{n=1}^{N}g_{n}^{2}\\ \;={\left|G(\theta )\right|}^{2}\exp\left[-(\sigma_{\varphi }^{2}+\sigma_{\lambda }^{2})\right]+(\sigma_{\varphi }^{2}+\sigma_{\lambda }^{2}+\sigma_{g}^{2}){\left\|\omega \right\|}^{2} \end{array}$$

where $\hat{G}\left(\theta \right)$ is the actual beampattern response. $\sigma_{g}^{}$ and $\sigma_{\varphi }^{}$ are the variances of mismatches in the amplitude and phase of weight coefficients. $\sigma_{\lambda }=(2\pi /\lambda )\cdot \sigma_{p}$, where $\sigma_{p}$ is the variance of the position mismatch of the antennas. $\left\|\cdot \right\|$ stands for the Euclidean *ℓ*_2_ norm. With constant mismatch variances, ${\left\|\omega \right\|}^{2}$ influences the sidelobes of the beampattern, thus, decreasing the robustness of the beamformer. In order to design a robust adaptive beamformer, the ${\left\|\omega \right\|}^{2}$ should be minimized, that is

(18) $$\underset{\omega }{\min}{\left\|\omega \right\|}^{2}$$

In engineering applications, much more clutter and many more moving targets exist on the ground than in the air. Thus, the echoes from the ground are much higher than those from the air. In order to mitigate the echoes from the ground clutter and ground moving targets at low altitude, the power emitted to the ground should be reduced. In the transmit beampattern, the sidelobes facing the ground side should be suppressed to a lower threshold, while the sidelobes facing the air side can be raised, as there are few interferences on the air side. The constraint on the sidelobe levels on the ground side can be written as

(19) $${\left|a^{H}\left(\theta \right)\omega \right|}^{2}\leq \delta ,\;\forall \theta \in \Theta_{G}$$

where $\Theta_{G}$ represents the set of locations on the ground side. δ is the prescribed sidelobe peak value for locations in $\Theta_{G}$ and can be specified to different values for different locations according to actual requirements.

When the constraints on the PAPR, robustness, and sidelobe levels are considered, the optimal TWV cannot be obtained directly by (11). To this end, we establish the following optimization problem:

(20) $$\begin{array}{l} \underset{\omega }{\min}\;\omega^{H}R\omega +{\left\|\omega \right\|}^{2}\\ s.t.\;\;a^{H}\left(\theta_{0}\right)\omega =1\\ \;\;N\frac{\underset{i=1,2,\ldots ,N}{\max}{\left|\omega_{i}\right|}^{2}}{{\left\|\omega \right\|}^{2}}\leq \zeta \\ \;\;{\left|a^{H}\left(\theta \right)\omega \right|}^{2}\leq \delta ,\;\forall \theta \in \Theta_{G}\; \end{array}$$

which can be interpreted as adaptive transmit beamforming under some practical array response constraints. The problem in (20) is a non-convex QCQP problem and cannot be solved in polynomial time.

Since $a^{H}\left(\theta_{0}\right)\omega =1$, we can obtain

(21) $$1={\left|a^{H}\left(\theta_{0}\right)\omega \right|}^{2}\leq {\left\|a\left(\theta_{0}\right)\right\|}^{2}{\left\|\omega \right\|}^{2}=N{\left\|\omega \right\|}^{2}$$

Therefore,

(22) $${\left\|\omega \right\|}^{2}\geq 1/N$$

Combining (22) into (20), we can obtain the upper bound of the PAPR:

(23) $$r_{\mathrm{PAPR}}=N\frac{\underset{i=1,2,\ldots ,N}{\max}{\left|\omega_{i}\right|}^{2}}{{\left\|\omega \right\|}^{2}}\leq N\frac{\underset{i=1,2,\ldots ,N}{\max}{\left|\omega_{i}\right|}^{2}}{1/N}=N^{2}\underset{i=1,2,\ldots ,N}{\max}{\left|\omega_{i}\right|}^{2}$$

The second constraint in (20) can be complemented by making the upper bound of the PAPR no more than ζ, that is,

(24) $$N^{2}\underset{i=1,2,\ldots ,N}{\max}{\left|\omega_{i}\right|}^{2}\leq \zeta$$

which can be equal to

(25) $$\underset{i=1,2,\ldots ,N}{\max}\left|\omega_{i}\right|\leq \sqrt{\zeta }/N$$

It can be assumed that

(26) $${\left\|\omega \right\|}^{2}=\omega^{H}\omega =\omega^{H}I_{N}\omega$$

The problem (20) can be converted to

(27) $$\begin{array}{l} \underset{\omega }{\min}\;\omega^{H}\left(R+I_{N}\right)\omega \\ s.t.\;\;a^{H}\left(\theta_{0}\right)\omega =1\\ \;\;\underset{i=1,2,\ldots ,N}{\max}\left|\omega_{i}\right|\leq \sqrt{\zeta }/N\\ \;\;\left|a^{H}\left(\theta \right)\omega \right|\leq \sqrt{\delta },\;\forall \theta \in \Theta_{G}\; \end{array}$$

where $I_{N}$ is an N×N identify matrix. The problem in (27) is a SOCP problem, which is a subclass of the convex programming problem. Optimizations can be performed with the program CVX [26], which is a high-level optimization routine that is based on MATLAB programming language.

Computational complexities: The complexities of the proposed approach mainly depend on the number of antenna elements *N*. The problem in (27) contains two linear matrix inequalities (LMIs) and one linear matrix equality (LME). Thus, in the interior-point method (IPM), the number of iterations required to reach the optimal solution is $O(\sqrt{N})$. For each iteration, the computational complexity is about $O(4N^{3})$. We know that the computational complexity of the conventional MVDR beamformer is $O(N^{3})$.

## 3. Simulations and Analyses

### 3.1. Experiment Settings

In this section, representative simulations are carried out to assess the effectiveness of the proposed, robust adaptive transmit beamforming scheme. As shown in Figure 1, a ULA with M=20 isotropy radiation elements spaced apart from each other by a half wavelength is considered. Considering the half wavelength array arrangement, it is required to ensure that the array element spacing is less than or equal to half of the wavelength. Considering that the signal is a narrowband signal, it needs to meet the following requirement: $B_{w}\leq 5\%f$, where $B_{w}$ means bandwidth, and f means carrier frequency. The beampattern is assumed to steer to $\theta_{0}=0^{\circ }$. Two interferences impinge on the array from the directions $-60^{\circ }$ and $20^{\circ }$, with the interference-to-noise ratios (INRs) 45 dB and 30 dB, respectively. The angular pattern covers $\left[-90^{\circ },90^{\circ }\right]$, with the sampling interval being $0.1^{\circ }$. The ground side includes the directions in the range of $\left[-90^{\circ },-5^{\circ }\right]$, while the air region is $\left[5^{\circ },90^{\circ }\right]$. The number of training snapshots is L=500. The optimizations are performed using the program CVX. Experiment settings are shown in Table 1.

To show the sidelobe quality, the indicators to measure the performance of sidelobe suppression are the peak sidelobe level (PSL) and the integrated sidelobe level (ISL). The PSL is the ratio of the maximum sidelobe power to the peak power. The ISL is the ratio of the total side lobe power to the peak power. The PSL and ISL are calculated as follows:

(28) $$PSL=10\log(\frac{\underset{\forall \theta \in \Theta_{G}}{\max}{\left|a^{H}(\theta )\omega \right|}^{2}}{{\left|a^{H}(\theta_{0})\omega \right|}^{2}})$$

(29) $$ISL=10\log(\frac{\int {\left|a^{H}(\theta )\omega \right|}^{2}}{{\left|a^{H}(\theta_{0})\omega \right|}^{2}}),\theta \in \Theta_{G}$$

The most basic DBF beamformer is the static beamformer. Its weighted value is the desired signal steering vector. Because it only has a phase shift, it is the simplest to implement and has a low sidelobe level. The static beamformer and SMI beamformer are realized as

(30) $$\left\{\begin{array}{c} \omega_{static}=a(\theta_{0})\\ \omega_{SMI}=\frac{R^{-1}a(\theta_{0})}{a(\theta_{0})R^{-1}a(\theta_{0})} \end{array}\right.$$

In this study, firstly, the beampattern of the proposed method is compared with classic beamformers in order to analyze the beampattern promotion. To show more details of the promotion and the comparisons of different beamformers on the transmit weight vector, zero depths are given in Table 2. Afterwards, to more intuitively understand the influence of the sidelobe level and the PAPR constraints on the beamformer, the parameter perturbation experiment is designed, and the simulation experiment is constructed under different parameter settings.

### 3.2. The Proposed Beampattern versus SMI and Static Beamformers

**Figure 2:**
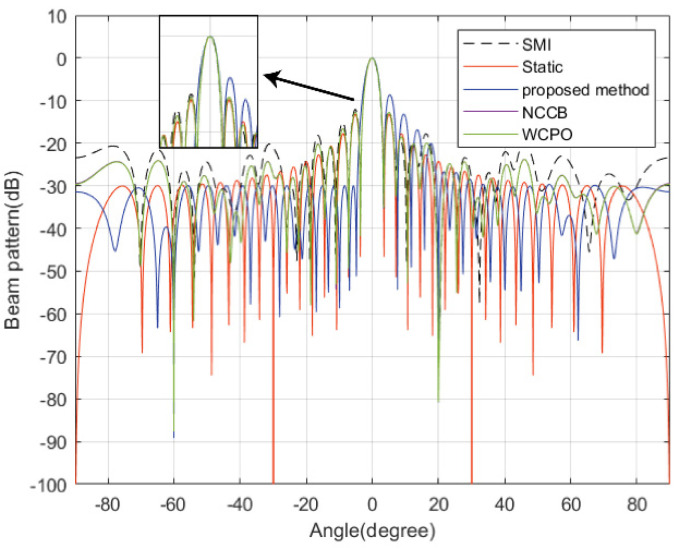
Beampatterns synthesized by different beamformers.

In this subsection, the beampattern of the proposed method is compared with the SMI beamformer, the static beamformer, norm-constrained Capon beamformer (NCCB), and worst-case performance optimization (WCPO), denoted by $\omega_{\mathrm{SMI}}$, $\omega_{0}=a\left(\theta_{0}\right)$, $\omega_{NCCB}$, and $\omega_{WCPO}$, respectively. In this example, the allowed PAPR is set to 1.2, while the sidelobes bound on the ground side are taken as −30 dB. Figure 2 shows the beams synthesized by different approaches. It can be clearly seen that sidelobes on the ground side obtained by the proposed method are lower than those achieved by the other two methods and do not exceed −30 dB. Table 2 displays the PAPR of the transmit weight vector, the zero depths in the interference directions, the PSLs of both the ground and air sides, and the ISLs of both the ground and air sides acquired by different methods with 1000 Monte Carlo trials carried out. It can be seen that the SMI beamformer can only form notches for interference suppression but meet neither sidelobe nor PAPR requirement. Therefore, the SMI beamformer cannot be used directly without further optimization. The static beamformer cannot form notches for interferences nor suppress sidelobes. NCCB and WCPO beamformers have high sidelobes, which result in greatly increased clutter. In contrast to the above methods, the presented approach can form notches in interference directions, effectively suppress the sidelobes on the ground side, and satisfy the PAPR requirement. The ISL comparisons show that the proposed method reduces the power emitted to the ground, while the power transmitting to the air is slightly increased.

### 3.3. The Beampattern under Different PAPR Tolerances

**Figure 3:**
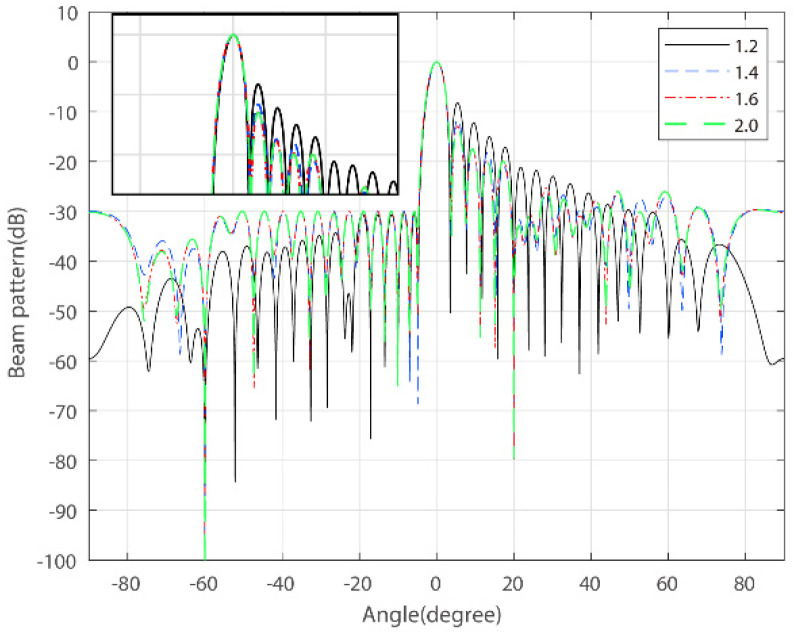
Beampatterns under the different PAPR upper bounds (sidelobe threshold = 30 dB).

In this example, the threshold for sidelobes on the ground side is still set to −30 dB, while the PAPR tolerance is set to 1.2, 1.4, 1.6, and 2.0. Figure 3 gives the beampatterns under different PAPR constraints. Table 3 illustrates the average performance with the actual PAPR, notch depths, PSL, and ISL for each PAPR upper bound. It can be noted that, with the relaxation of the PAPR constraint, the sidelobe growth on the air side is not as significant. The PAPR threshold can be selected according to practical system requirements.

### 3.4. The Beampattern under Different Sidelobe Thresholds

**Figure 4:**
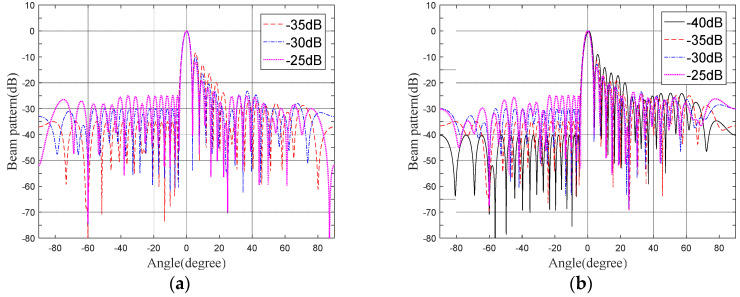
Beampatterns under the same PAPR upper bound and different sidelobe thresholds. (**a**) PAPR upper bounds = 1.2. (**b**) PAPR upper bounds = 1.4.

The sidelobe threshold $\rho_{s}$ is set to −25 dB, −30 dB, −35 dB, and −40 dB, with the PAPR bound $\rho_{p}$ being 1.2 and 1.4. When $\rho_{s}=-40\mathrm{dB}$ and $\rho_{p}=1.2$, the restrictions are so strict that the objective problem cannot be solved. Figure 4a,b shows the beampatterns under different sidelobe suppression requirements, with $\rho_{p}$ being 1.2 and 1.4. We can see that the presented method meets all design requirements, except for the overly strict constraints. It can be noted that, with the strictness of the sidelobes bound on the ground side, it causes a rise in the sidelobe level on the air side.

## 4. Conclusions

To block interference suppression, e.g., ground, a robust adaptive transmit beamforming under the constraint of a low PAPR is proposed in this paper. In this approach, we allow the sidelobe to face the ground under the threshold so that strong ground jamming is suppressed. The constraint on the PAPR of the TWV is used to avoid excessive energy attenuation. We then give the final form of this optimization model. By transforming the optimization model into an SOCP problem, it can be solved easily using the CVX toolbox. Simulation results show that the proposed method well suppresses the interference on the ground side and air side. The sidelobe level of the ground side is low, and the excess signal energy is transmitted to the air side. By employing the proposed method, the detection performance of the radar can be effectively improved.

## Figures and Tables

**Table 1:** Experiment settings.

Array Type	ULA	Interference Angle2	$\theta_{2}=20^{\circ }$
$\frac{d}{\lambda }$	0.5	INR1	45 dB
*N* elements	20	INR2	30 dB
Expected signal	$\theta_{0}=0^{\circ }$	Snapshots	500
Interference angle1	$\theta_{1}=-60^{\circ }$	Ground side	$\left[-90^{\circ },-5^{\circ }\right]$

**Table 2:** Comparisons on the performances of the PAPR, zero depth, PSL, and ISL for different methods.

Method	Actual PAPR	Zero Depth (dB)			PSL (dB)	ISL (dB)	
		−60°	20°	Ground	Air	Ground	Air
SMI	1.8241	−87.5525	−72.6318	−13.1341	−13.1067	5.1249	5.2802
Static	1	−37.1202	−24.3174	−13.2360	−13.2360	2.0249	2.0249
NCCB	1.3642	−88.11	−92.88	−12.9856	−14.7296	6.7218	4.7574
WCPO	1.3592	−88.12	−93.06	−12.9923	−14.7051	6.6584	4.7186
Proposed Method	1.0674	−87.4107	−72.3739	−30.0467	−8.4458	−5.5559	6.7245

**Table 3:** Comparisons on the performances of the PAPR, zero depth, PSL, and ISL for different PAPR upper bounds.

PAPR Upper Bound	Actual PAPR	Zero Depth (dB)		PSL (dB)		ISL (dB)	
		−60°	20°	Ground	Air	Ground	Air
1.2	1.0529	−96.4369	−79.2629	−30.1025	−8.2828	−8.3146	7.1537
1.4	1.3009	−96.9545	−81.6837	−30.0000	−11.5818	−4.3383	3.6278
1.6	1.4904	−96.8575	−81.8493	−30.0000	−12.9159	−4.2702	2.8470
2.0	1.5886	−96.7653	−81.7726	−30.0000	−13.0978	−4.2742	2.7777

## Data Availability

Not applicable.
